# Characteristics and outcome of mechanically ventilated patients with 2009 H1N1 influenza in Bosnia and Herzegovina and Serbia: impact of newly established multidisciplinary intensive care units

**DOI:** 10.3325/cmj.2012.53.620

**Published:** 2012-12

**Authors:** Marija Kojičić, Pedja Kovačević, Nermina Bajramović, Uroš Batranović, Jadranka Vidović, Kenana Aganović, Srdjan Gavrilović, Biljana Zlojutro, Guillaume Thiery

**Affiliations:** 1 Medical Intensive Care Unit, The Institute for Pulmonary Diseases of Vojvodina, Sremska Kamenica, Serbia; 2 Medical Intensive Care Unit, Clinical Center University of Banja Luka, Bosnia and Herzegovina; 3 Medical Intensive Care Unit, Clinical Center University Sarajevo, Bosnia and Herzegovina; Kojičić et al: Characteristics and outcome of mechanically ventilated patients with 2009 H1N1 influenza in BH and Serbia

## Abstract

**Aim:**

To describe characteristics and outcome of mechanically ventilated patients admitted to three newly established intensive care units (ICU) in Bosnia-Herzegovina and Serbia for 2009 H1N1 influenza infection.

**Methods:**

The retrospective observational study included all mechanically ventilated adult patients of three university-affiliated hospitals between November 1^,^ 2009 and March 1 2010 who had 2009 H1N1 influenza infection confirmed by real-time reverse transcriptase-polymerase-chain-reaction (RT-PCR) from nasopharyngeal swab specimens and respiratory secretions.

**Results:**

The study included 50 patients, 31 male (62%), aged 43 ± 13 years. Median time from hospital to ICU admission was 1 day (range 1-2). Sixteen patients (30%) presented with one or more chronic medical condition: 8 (16%) with chronic lung disease, 5 (10%) with chronic heart failure, and 3 (6%) with diabetes mellitus. Thirty-two (64%) were obese. Forty-eight patients (96%) experienced acute respiratory distress syndrome (ARDS), 28 (56%) septic shock, and 27 (54%) multiorgan failure. Forty-five patients (90%) were intubated and mechanically ventilated, 5 received non-invasive mechanical ventilation, 7 (14%) high-frequency oscillatory ventilation, and 7 (14%) renal replacement therapy. The median duration of mechanical ventilation was 7 (4-14) days. Hospital mortality was 52%.

**Conclusion** Influenza 2009 H1N1 infection in three southeast European ICUs affected predominantly healthy young patients and was associated with rapid deterioration after hospital admission and severe respiratory and multiorgan failure. These emerging ICUs provided contemporary ICU services, resulting in case-fatality rate comparable to reports from well-established ICU settings.

After the first cases of human influenza A H1N1 infection were observed in Mexico in April 2009 (1), the infection quickly spread to Europe, Asia, and both Americas, which is why the World Health Organization (WHO) declared level 6 pandemic alert on June 11, 2009 (2). Up to February 2010, influenza A H1N1 virus was confirmed in more than 212 countries and caused more than 15 000 deaths (3).

Compared with seasonal influenza, pandemic 2009 H1N1 influenza A has affected a great number of young, previously healthy patients and caused significant health and economic burden even in developed countries. So far, several studies have reported their experience with critically ill patients with 2009 H1N1 influenza in North America, South America, Asia, Australia, and Europe (1,4-12). A few studies have so far reported on H1N1 patients' characteristics and outcome in low-income and developing countries with limited ICU resources (1,8-12), and none on that in the west Balkans. The ICUs in these countries are mainly run by physicians who lack formal ICU training. Furthermore, until only recently non-surgical critically ill patients in these ICUs had limited access to mechanical ventilation and other life support interventions. However, over the past five years, three university hospitals in Bosnia and Herzegovina and Serbia have established multidisciplinary medical ICUs and most of critically ill patients with H1N1 influenza A infection in the region were admitted to these units.

The aim of this study was to describe characteristics and outcomes of mechanically ventilated adult patients with 2009 H1N1 influenza A infection treated in these ICUs.

## Materials and methods

A retrospective observational study was conducted in the ICUs of three university-affiliated hospitals: Clinical Center of University in Sarajevo (5 beds, medical ICU), University Hospital of Banja Luka, Banja Luka (6 beds, medical ICU) (Bosnia and Herzegovina), and the Institute for Pulmonary Diseases of Vojvodina, Sremska Kamenica (5 beds, medical ICU) (Serbia) between November 1, 2009 and March 1, 2010. All three ICUs have been established in the last five years (Sarajevo in 2009, Banja Luka in 2008, and Sremska Kamenica in 2003) with support of critical care specialists trained in the United States and Europe, members of the European Society of Intensive Care Medicine, and the Society of Critical Care Medicine (13).

The study included all mechanically ventilated patients with 2009 influenza A(H1N1) virus infections. Patients had novel Influenza A (H1N1) infection confirmed by real-time reverse transcriptase polymerase chain reaction (RTC-PCR) from nasopharyngeal swab specimens and respiratory secretions at the time of hospital admission. Data were extracted from pre-existing hospital charts including demographic data, vital signs, laboratory parameters, radiological data, mechanical ventilation details, and chronic medical conditions: chronic obstructive pulmonary disease, asthma, congestive heart failure, neoplasm, chronic liver or renal diseases, diabetes mellitus, and the use of immunosuppressant medications. Severity of illness was assessed according to the Acute Physiology and Chronic Health Evaluation (APACHE) II score (14). Standardized definitions were used to determine the presence or absence of ICU complications including acute respiratory distress syndrome (ARDS) (15) and shock (16). The study protocol was approved by the Institutional Review Boards, which waived the need for additional informed consent due to an observational study design.

### Statistical analyses

Data are expressed as the mean and standard deviation for normally distributed or as median and interquartile range for not normally distributed continuous variables, and counts with percentages for categorical variables. Comparison of measures for continuous variables was done using Wilcoxon rank sum test. Comparison of proportion was done using χ^2^ square test and Fisher exact test as appropriate. Statistical analyses were performed using JMP statistical software (JMP 7, SAS institute, Cary, NC, USA). *P*-values <0.05 were considered statistically significant.

## Results

### Clinical characteristics

A total of 50 patients, 31 men (62%), mean age 43 ± 13 years, were admitted to medical ICUs of three tertiary care hospitals (Table 1). Mean Apache II score was 19 ± 11. The most frequent comorbidities were obesity, smoking, and chronic respiratory disease (Table 1). Two thirds of patients did not have any chronic medical condition. The median time from hospital admission to ICU admission was 1 day (1-2 days). Antiviral treatment was initiated on average six days after symptom onset.

**Table 1:** Characteristics of mechanically ventilated patients with 2009 influenza A(H1N1) confirmed infection*

Characteristic	
Age, median (range), y	43 (35-54)
Male sex, n (%)	31 (62)
APACHE II (mean ± standard deviation)	19 ± 11
SOFA on day 1 (mean ± standard deviation)	8 ± 4
Chronic medical conditions, n (%)^†^	16 (32)
Obesity (BMI>30), n (%)	32 (64)
Ever smoker, n (%)	14 (28)
Chronic respiratory disease, n (%)	8 (16)
Congestive heart failure, n (%)	5 (10)
Diabetes, n (%)	3 (6)
Immunosuppression, n (%)	3 (6)
Pregnancy, n (%)	4 (8)

*Abbreviations: APACHE – Acute Physiology and Chronic Health Evaluation; SOFA – Sequential Organ Failure Assessment; BMI – body mass index.

^†^Indicates presence of chronic respiratory diseases, congestive heart failure, diabetes, immunosupresion, malignancy, chronic renal or liver disease.

### Respiratory failure

Forty-seven patients (94%) presented with bilateral infiltrates on chest radiographs at the ICU admission and 48 patients (96%) developed ARDS. The median P/F ratio (arterial oxygen concentration to the fraction of inspired oxygen) on day 1 was 75 mm Hg (interquartile range, 57-129). The proportion of patients with bilateral infiltrate was similar among survivors and non survivors. However the P/F ratio was significantly lower among non survivors (121 vs 59, *P* < 0.001) (Table 2). Forty-five patients (90%) were intubated and mechanically ventilated and 8 received tracheostomy. Five patients were treated with non-invasive mechanical ventilation only, and all of them survived. High frequency oscillation was used in 7, and renal replacement therapy in 7 patients.

**Table 2:** Characteristics of survivors and non survivors on intensive care unit (ICU) admission

	Survivors (N = 24)	Non-survivors (N = 26)	*P*
**Baseline characteristics**			
Age (median, IQR), y	42 (32-53)	43 (35-55)	0.816^†^
Male sex, n (%)	16 (52)	15 (48)	0.514^‡^
Ever smoker, n (%)	7 (29)	7 (27)	0.860^‡^
BMI (median, IQR)	30 (27-32)	30 (26-34)	0.881^†^
Any comorbidities, n (%)	7 (29)	9 (35)	0.680^‡^
Chronic pulmonary disease, n (%)	4 (17)	4 (15)	1.000^§^
CHF, n (%)	2 (8)	3 (12)	1.000^§^
Diabetes mellitus, n (%)	2 (9)	1(4)	0.602^§^
Immunosupression, n (%)	0 (0)	3 (12)	0.236^§^
Pregnancy, n (%)	1 (4)	3 (12)	0.610^§^
**ICU admission characteristics**			
Apache II^a^ (median, IQR)	13 (5-18)	24 (15 -31)	<0.001^†^
SOFA day 1^a^ (median, IQR)	5 (3-8)	8 (7-13)	<0.001^†^
GCS^a^ (median, IQR)	15 (15-15)	10 (8-15)	0.001^†^
Temperature (°C)^b^ (median, IQR)	38 (37-39)	39 (37-39)	0.741^†^
Heart rate^a^ (median, IQR)	108 (100-130)	129 (113-141)	0.011^†^
Systolic blood pressure (mmHg) (median, IQR)	110 (98-130)	90 (82-116)	0.018^†^
Respiratory rate (per min) (median, IQR)	26 (20-38)	36 (30-45)	0.008^†^
Pao_2_/FiO2 d 1^b^, (median, IQR)	121(76-199)	59 (50-80)	<0.001^†^
WBC count^b^ 10^9^/L, (median, IQR)	6 (5-7)	4 (2-8)	0.077^†^
Hct^b^, (median, IQR)	35 (32-47)	34 (28-32)	0.594^†^
Platelet count^b^ 10^3^/μL, (median, IQR)	185 (133-224)	127 (89-168)	0.077^†^
Urea^b^ mmol/L, (median, IQR)	5 (3-7)	8 (5-13)	0.016^†^
Creatinin^b^ mg/dL, (median, IQR)	78 (69-99)	106 (84-181)	0.002^†^
Bilirubin^b^ μmol/L, (median, IQR)	9 (7-15)	14 (7-19)	0.376^†^
Bilateral infiltrates, n (%)	21 (87)	26 (100)	0.103^§^

*Abbreviations: BMI – body mass index; CHF – congestive heart failure; APACHE – Acute Physiology and Chronic Health Evaluation; SOFA- Sequential Organ Failure Assessment; GCS-Glasgow Coma Scale; IQR – interquartile range;^a^ at the time of ICU admission, ^b^ worst value on day 1.

†Wilcoxon rank sum test.

‡χ^2^ test.

§Fisher exact test.

### Non pulmonary organ failure

Fourteen (28%) patients required vasoactive medications on day 1, but 28 (56%) eventually developed septic shock and 27 (54%) multiorgan failure. Six (12%) patients required hemodialysis. Hemodynamic, neurological, and renal impairment were more frequent among non-survivors (Table 2).

### Outcomes

The median duration of mechanical ventilation was 7 days (4-14 days) and median ICU length of stay was 10 days (5-22 days). Overall hospital mortality was 52% (n = 26). The mortality rates were higher in pregnant (75%, 3/4) and obese patients (53%, 17/32). The time from the symptom onset to administration of oseltamivir was longer in non-survivors than survivors, although the difference was not significant. Higher severity of illness scores and hypoxemia on admission were strongly associated with increased mortality (Table 2, Table 3).

**Table 3:** Interventions and outcomes among survivors and non survivors

	Survivors (N = 24)	Non-survivors (N = 26)	*P*
**Interventions and complications**			
Time to oseltamivir, days (median, IQR)	5 (4-8)	6 (5-7)	0.234^†^
ARDS, n (%)	22 (92)	26 (100)	0.225^‡^
Septic shock, n (%)	5 (21)	23 (88)	<0.001^§^
MODS, n (%)	9 (43)	18 (95)	<0.001^§^
Dialysis, n (%)	1 (4)	6 (23)	0.100^‡^
Neuromuscular blockers, n (%)	7 (30)	14 (54)	0.077^§^
High frequency oscillatory ventilation, n (%)	0 (0)	7 (27)	0.010^‡^
Tracheostomy, n (%)	5 (21)	3 (11)	0.456^‡^
Ventilator associated pneumonia, n (%)	8 (33)	12 (46)	0.355^§^
**Outcomes**			
Mechanical ventilation, days (median, IQR)	7 (4-10)	10 (4-18)	0.572^†^
ICU length of stay, days (median, IQR)	10 (7-33)	10 (4-18)	0.158^†^

*Abbreviations: ARDS – acute respiratory distress syndrome; MODS – multiple organ dysfunction syndrome; ICU – intensive care unit; IQR – interquartile range.

†Wilcoxon rank sum test.

‡χ^2^ test.

§Fisher exact test.

## Discussion

We report on the first series of critically ill patients from southeastern Europe admitted to 3 newly established ICUs for confirmed influenza A (H1N1) infection requiring mechanical ventilation. Patients were mostly young and most of them did not have any chronic medical condition. Obesity was the most frequently associated comorbidity. All patients presented with severe hypoxemic respiratory failure and most of them with hemodynamic failure. In terms of patients' characteristics, our results are consistent with previously published data (1,4-12).

In the west Balkans, the outbreak of the novel influenza virus began in the late fall of 2009, and its impact was assessed during the winter flu season. Similar to previous studies, we observed a higher percentage of critically ill among young, previously healthy patients than was the case with seasonal influenza (1,4-12,17). The most common chronic medical conditions were chronic lung diseases and 30% of patients had a history of smoking. Although obesity was common, there was no difference in body mass index between survivors and non-survivors. Patients experienced rapid deterioration following hospitalization and presented with ARDS, often combined with septic shock and multiorgan failure, with a great need for rescue therapies such as renal replacement therapy of high-frequency oscillatory ventilation. There was a high occurrence of complications such as prolonged mechanical ventilation and ventilator-associated pneumonia. Inhaled nitric oxide and extracorporeal membrane oxygenation were not available. The number of days on mechanical ventilation and average length of stay were similar to other studies (4,7).

Previous studies reported on young patients with very severe critical illness (Table 4). Obesity was the most common comorbidity, ranging from 21% to 64% in all patients. Although there were some discrepancies between the studies, the number of patients with no comorbidities was substantial, ranging from 15% to 64%, except in one study (4), in which it was only 1.7%. The pattern of the respiratory failure was similar across the studies as most patients developed a severe form of ARDS. The level of hypoxemia was high in all studies. Hemodynamic failure was also frequently present, which is consistent with previously published data (1,6,9,10,12). In our study, the majority of patients were treated with invasive mechanical ventilation. In other studies, this proportion ranged from 1% (1) to 19% (9). Mortality rates found in our study were similar to those from other studies, ranging between 40% and 50% in the majority of the studies. Only a Canadian study (4) reported a 21% mortality rate, whereas a South African study reported a 68% mortality rate (10). The mortality rate in our study was comparable to those in other transitional countries (1,8,9), but higher than in some reports from Western countries, with longer tradition of intensive care medicine (4,6,7,18). The high morbidity and mortality rate in patients with confirmed 2009 H1N1 influenza imposed significant burden even on well developed countries and advanced ICU settings. Many previous reports (19-21) emphasized the potential problem of ICU's unpreparedness for the upcoming pandemic. Physicians in Bosnia and Herzegovina and Serbia, as well as in the majority of countries in Eastern Europe, lack official ICU training (13) and use annual meetings and conferences as sources of information. This all affects the quality and availability of the ICU resources, especially in cases when there is a need for advanced rescue therapies. The contemporary ICUs are available in a small number of centers throughout the region, with restricted number of beds, and when faced with increased demands, the consequences can be devastating. In Bosnia and Herzegovina and Serbia, the 2009 winter outbreak had occurred before massive vaccination was introduced and appropriate antiviral therapy in severely ill patients during the epidemic was generally delayed.

**Table 4:** Comparison of studies reporting characteristics and outcome of patients with influenze A(H1N1) virus infection requiring mechanical ventilation

	Canada (4)	Mexico (1)		Argentina (9)	Uruguay(8)	South Africa (10)	India (12)	Spain (6)	Southeastern Europe (our data)
Patients, N	16	58		337	96	19	31	32	50
Ventilated patients, n (%)	136 (81)	54 (93)		337 (100)	96 (100)	19 (100)	22 (71)	24 (75)	50 (100)
**Epidemiology and risk factors:**									
H1N1 confirmed, n (%)	162 (94)	29 (50)		132 (39)	77 (80)	NA	31 (100)	32 (100)	50 (100)
age, y (mean±SD or median and range)	32 ± 21	44 (10-83)		47 ± 17	45 ± 14	39.5 ± 14.8	35 (28-43)	36 (31-52)	43 (35-54)
female/male (ratio)	67/33	53/37		44/56	44/56	79/21	42/58	27/73	38/62
APACHE II, mean ± SD	19.7 ± 8.7	20.1 ± 11.9		18 ± 7	18 ± 8	18 ± 5	13.9 ± 6.9	13.8 ± 6.4	18.7 ± 10.6
SOFA, mean ± SD	6.8 ± 3.6	9 ± 4.3		NA	5.8 ± 2.2	NA	NA	7.1 ± 3.3	7.7 ± 3.7
no comorbidity, n (%)	3 (1.7)	9/58 (15)		121 (36)	27 (24)	3 (16)	20 (35)	15 (47)	34 (68)
diabetes, n (%)	35 (21)	10 (17)		41 (12)	14 (15)	6 (32)	3 (9.7)	1 (3)	3 (6)
immunosuppression, n (%)	33 (20)	2 (3.4)		50 (15)	8 (8)	6 (32)	1 (3.2)	NA	3 (6)
COPD, n (%)	16 (9.5)	2 (3.4)		61 (18)	17 (17)	1 (5)	1 (3.2)	4 (12.5)	6 (12)
obesity, n (%)	56 (33.3)	21 (36)		80 (24)	37 (39)	4 (21)	9 (29)	10 (31)	32 (64)
pregnancy, n (%)	13 (7.7)	1 (1.7)		22 (7)	6 (6)	6 (32)	3 (9.7)	2 (6)	3 (6)
**Time periods:**									
time from onset to hospitalization, days (median and IQR)	4 (2-7)	6 (4-8)		6 (3-8)	4 (2-7)	NA	NA	NA	NA
time from hospitalization to ICU, days (median and IQR)	1 (0-2)	1 (0-3)		0 (0-2)	0 [0-2]	NA	NA	NA	1 (1-2)
time from symptoms onset to ICU, days (median and IQR)	NA	NA		NA	NA	NA	6(5-7)	3 (2-6)	NA
**Ventilation parameters:**									
Pao_2_/FiO2 on admission (mmHg, mean±SD or median and range)	147 ± 128	83 (59-145)		107 (75-150)	116 (73-220)	171 ± 74	NA	NA	103 ± 63
Pao_2_/FiO2 < 100	NA	NA		151 (45)	NA	3 (16)	12 (38.7)	NA	NA
NIV only	8 (5)	6 (1)		64 (19)	10 (10.4)	2 (10)	4 (12.9)	2 (6)	5 (10)
prone	5 (3)	NA		43 (13)	25 (26)	NA	9 (40.9)	8 (25)	2 (4)
ECMO	7 (4.2)	0		0	7	0	0	0	0
**Other organ failures and treatment:**									
shock (or vasopressors) on admission, n (%)	55 (33)	34 (58.6)		NA	NA	NA	NA	NA	14 (28)
shock (or vasopressors) during the ICU stay, n (%)	NA	NA		242 (72)	NA	8 (42)	18 (58.1)	30 (62)	28 (56)
corticosteroids, n (%)	85 (50.1)	40 (69)		NA	53 (55)	2 (10)	NA	NA	NA
renal replacement therapy, n (%)	NA	NA		55 (17)	NA	3 (16)	4 (12.9)	7 (22)	7 (14)
**Outcome:**									
duration of mechanical ventilation (median, IQR)	12 (6-20)	15 (8-26) S 3 (3-13) nonS	10 (5-16)		9 (2-14) S 9 (4-16) nonS	NA	10 (4-22)	10 (1-21) S	7 (4-14)
length of stay in the ICU (median, IQR)	12 (5-20)	13.5 (6-24) S 7 (2-13) Non S	12 (6-20)		10 (4-19) S 10 (4-16) Non S	15 ± 11	10 (5-17)	NA	10 (5-22)
overall mortality, n (%)	29 (17.3)	24(41.4)	156 (46)		48 (50)	13 (68)	6 (19.4)	8/32 (25^†^)	26 (52)
mortality in mechanically ventilated patients, n (%)	29 (21)	24(44.4)	156 (46)		48 (50)	13 (68)	NA	8/24 (33^†^)	26 (52)

*Abbreviation: S – survivors; non S – non-survivors; SD – standard deviation; APACHE – Acute Physiology and Chronic Health Evaluation; SOFA – Sequential Organ Failure Assessment; COPD – chronic obstructive pulmonary disease; ICU – intensive care unit; NIV – noninvasive mechanical ventilation; ECMO – extracorporeal membrane oxygenation; IQR – interquartile range.

†28-d mortality. 2 more deaths at day 31 and 65, + at day of submission, 5/28 patients were still ventilated.

It is noteworthy that all 3 ICUs have been set up recently, one of them had opened 3 months before the breakout of the pandemic, with poorly trained staff and inappropriate equipment (infusion pumps were not available in one ICU during the whole study period and renal replacement therapy was inconstantly possible to perform). In spite of all this, half of the patients survived.

Intensive care medicine is frequently considered to have lower priority in low and middle income countries (22-24). Although costs of intensive care are high, population based studies have proven it “very cost effective” according to WHO definition (25-27).

The limitations of our study are related to a relatively small number of patients. The study included patients from three tertiary care centers who required mechanical ventilation and ICU admission and underwent routine testing for H1N1 infection the flu season. This may not necessarily be representative of all regional centers, and the total burden of pandemic in the region is likely to be higher. The study was performed on the patients with the most severe form of disease, requiring intensive care and is not representative of less severe cases who did not require admission to the ICU. Finally, high frequency oscillation was available only in one center and some rescue therapies such as extracorporeal membrane oxygenation and inhaled nitric oxide were not accessible at all.

The novel 2009 H1N1 influenza in Bosnia and Herzegovina and Serbia showed similar characteristics to those reported in other studies from around the world, affecting predominantly young healthy individuals and those with no comorbidities. Patients presented with severe ARDS, had a great need for rescue therapies, and a high mortality. In an area with low access to intensive care and physicians’ lack of training in intensive care medicine, these three newly established ICUs achieved a survival rate similar to that in other transitional countries, and slightly lower than in developed countries.
